# A Novel Marine Natural Product Derived Pyrroloiminoquinone with Potent Activity against Skin Cancer Cells

**DOI:** 10.3390/md17080443

**Published:** 2019-07-27

**Authors:** Jaden Cowan, Mohammad Shadab, Dwayaja H. Nadkarni, Kailash KC, Sadanandan E. Velu, Nabiha Yusuf

**Affiliations:** 1 Department of Chemistry, University of Alabama at Birmingham, 901, 14th Street S, Birmingham, AL 35294, USA; 2 Department of Dermatology, University of Alabama at Birmingham, 1670 University Boulevard, VH 566A, P.O. Box 202, Birmingham, AL 35294, USA; 3 Veteran Affairs Medical Center, Birmingham, AL 35294, USA

**Keywords:** pyrroloiminoquinone, alkaloid, analog, skin cancer, apoptosis, SCC13, invasion, migration

## Abstract

Non-melanoma skin cancer is one of the major ailments in the United States. Effective drugs that can cure skin cancers are limited. Moreover, the available drugs have toxic side effects. Therefore, skin cancer drugs with less toxic side effects are urgently needed. To achieve this goal, we focused our work on identifying potent lead compounds from marine natural products. Five lead compounds identified from a class of pyrroloiminoquinone natural products were evaluated for their ability to selectively kill squamous cell carcinoma (SCC13) skin cancer cells using an MTT assay. The toxicity of these compounds was also evaluated against the normal human keratinocyte HaCaT cell line. The most potent compound identified from these studies, **C278** was further evaluated for its ability to inhibit cancer cell migration and invasion using a wound-healing assay and a trans-well migration assay, respectively. To investigate the molecular mechanism of cell death, the expression of apoptotic and autophagy proteins was studied in **C278** treated cells compared to untreated cells using western blot. Our results showed that all five compounds effectively killed the SCC13 cells, with compound **C278** being the most effective. Compound **C278** was more effective in killing the SCC13 cells compared to HaCaT cells with a two-fold selectivity. The migration and the invasion of the SCC13 cells were also inhibited upon treatment with compound **C278**. The expression of pro-apoptotic and autophagy proteins with concomitant downregulation in the expression of survival proteins were observed in **C278** treated cells. In summary, the marine natural product analog compound **C278** showed promising anticancer activity against human skin cancer cells and holds potential to be developed as an effective anticancer agent to combat skin cancer.

## 1. Introduction

Skin cancer is one of the most frequently found cancers in humans, especially in the United States [1,2]. The three main types of skin cancer are basal cell carcinoma (BCC), squamous cell carcinoma (SCC) and melanoma [3]. Out of these, BCC and SCC are more common and are referred to as non-melanoma skin cancers (NMSCs) [2]. Incidence of skin cancer is still increasing due to factors such as increased UV exposure [4], disturbance in the environmental conditions [5] and hereditary risk factors [6,7]. Although surgical methods remain the most common form of treatment for non-melanoma skin cancers, there is still a demand for affordable and less toxic treatment options [6].

Some of the recent efforts aimed at developing skin cancer treatments were focused on quinone-based compounds that have shown potent cell killing activities against skin cancer cells [7,8,9]. For instance, a penanthroperylenequinone, hypericin, one of the active components of St. John’s Wort (*Hypericumperforatum*) has been found to be activated by UVA and induced melanoma cell death through the processes of apoptosis and necrosis [10]. Other compounds such as triterpene-quinone fraction isolated from the roots of *Ardisiacrispa* has inhibited the chemical-induced mouse skin tumor promotion [7]. Thus, quinone-based small molecule compounds hold potential to be developed as anti-skin cancer agents. One of the mechanisms of action of the quinone compounds relies on its reactivity with cellular glutathione (GSH). Quinone moiety reacts spontaneously with GSH to produce GS-hydroquinones. These hydroquinones are readily auto-oxidized to form semiquinones, superoxide anion (O_2_^•−^) and H_2_O_2_ [11]. As a result, cellular GSH levels are reduced and the cellular level of reactive oxygen species (ROS) is increased by manifold. Both of these conditions are devastating factors to the cells leading to cell death [12]. 

**Figure 1:**
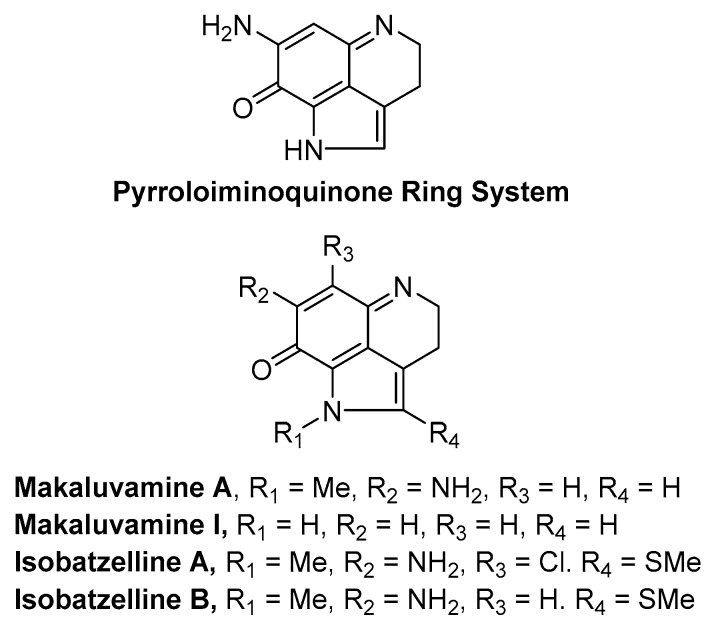
Representative examples of marine natural products containing the pyrroloiminoquinone ring system.

Considering the significance of the quinone compounds in skin cancer therapy, we focused our research on identifying potent quinone-based lead compounds from marine natural products. For more than half a century, global marine sources have proven to be a rich source of vast array of new medicinally valuable compounds [13,14,15,16]. Marine sponges produce a plethora of chemical compounds with widely varying carbon skeletons. Most bioactive compounds from sponges have exhibited a variety of activities such as anticancer, anti-inflammatory and antibiotic activities. Marine sponges of the genera Latrunculia, Batzella, Prianos and Zyzzya are a rich source of alkaloids bearing a pyrrolo[4,3,2-de]quinoline skeleton [17,18]. Their unique fused ring skeletons carry interesting biological properties making them targets for several synthetic and biological studies. Our interest is focused on pyrroloiminoquinone alkaloids which belong to this family. Makaluvamines are a group of pyrroloiminoquinone alkaloids isolated from four species of marine sponges, namely the Fijian sponge *Zyzzya* cf. *marsailis* [19], Indonesian sponge *Histodermella* sp. [20], Pohnpeian sponge *Zyzzya fuliginosa* [21,22,23] and Jamaican sponge *Smenospongia aurea* [24]. A few examples of pyrroloiminoquinone alkaloids are presented in Figure 1.

**Figure 2:**
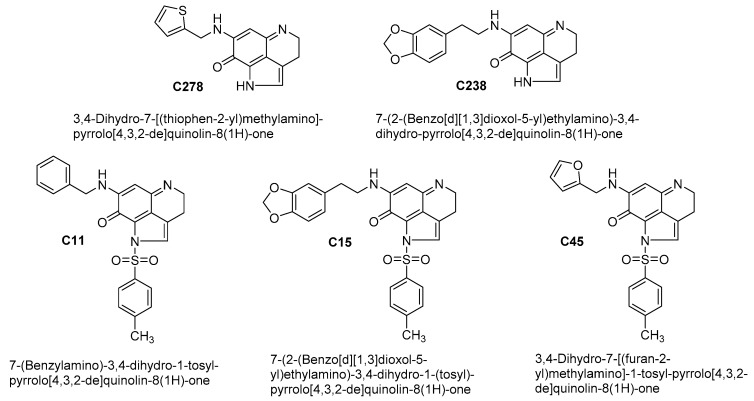
Chemical structures of the test compounds, **C278**, **C238**, **C11**, **C15** and **C45**.

We have extensively investigated the anticancer activities of pyrroloiminoquinone alkaloid analogs in breast, prostate, ovarian, liver and pancreatic cancers in the past decade [25,26,27,28,29,30,31]. We have also recently developed a hydrogel based drug-delivery platform for one of the lead compounds identified from these studies [32]. However, the activity of these compounds against skin cancer has not been evaluated yet. This manuscript describes our initial findings on the synthesis and evaluation of the lead compound (**C278**, Figure 2) identified from our initial *in vitro* screening of marine alkaloid analogs against non-melanoma cell line SCC13. In this report, we provide evidence to show that compound **C278** kills non-melanoma skin cancer cells very effectively. In addition, compound **C278** was found to inhibit migration and invasive properties of the SCC13 cells. A mechanistic study revealed that **C278** induces apoptosis and autophagy in SCC13 cells. Taken together, this study suggests that newly synthesized marine natural product derived quinone compound **C278** holds promise to be developed as an effective anticancer agent for skin cancer therapeutic intervention.

## 2. Results

### 2.1. Alkaloid Analogs Induce Killing of Human Skin Cancer Cells in a Dose-Dependent Manner

We first investigated the dose-dependent effects of all five compounds on the cell viability of human skin cancer cell line, SCC13 as well as an immortalized human skin keratinocyte cell line, HaCaT through MTT assay. The cells were treated for 24 h with increasing doses of the compounds ranging from 0.0625 µM to 1.0 µM and found that the viability of the cells was inhibited in a dose-dependent manner. IC_50_ and IC_90_ values for these compounds against these two cell lines were calculated (Table 1). There were statistically significant differences between these two values with *p* < 0.05.

**Figure 3:**
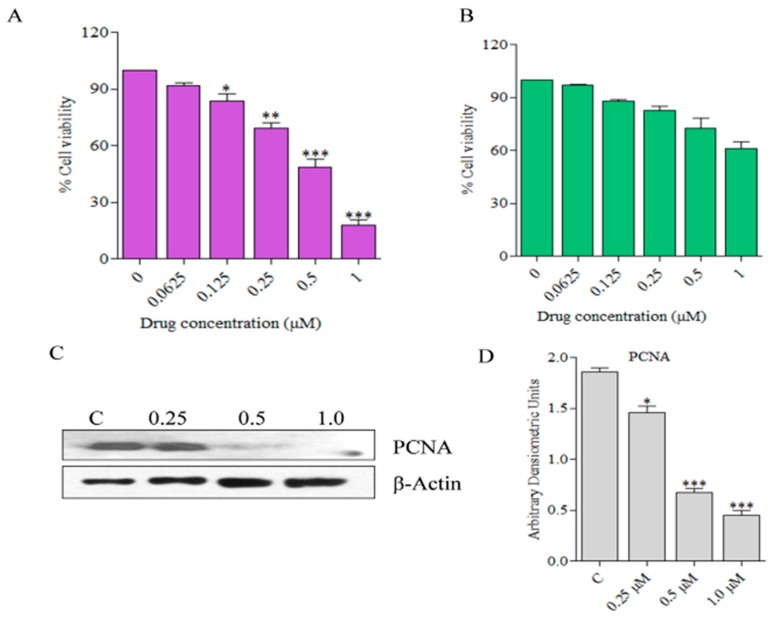
Effects of **C278** on the viability of SCC13 and HaCaT cells (**A**) SCC13 cells were treated with increasing concentrations of **C278** at 24 h, and the percentage cell viability was estimated. Results are combined from two independent experiments and are presented as mean ± SD; *n* = 4. (**B**) HaCaT cells were treated with increasing concentrations of **C278** at 24 h, and the percentage cell viability was estimated. Results are combined from two independent experiments and are presented as mean ± SD; *n* = 4. (**C**) Expression of PCNA was measured through western blot. The expression of this protein was normalized using β-Actin as a loading control. Figures are representatives of 2 independent experiments. (**D**) Densiometric analysis of bands was performed and the bar graphs expressing arbitrary units are presented. Error bar represents mean ± SD, *n* = 4. * *p* < 0.01, ** *p <* 0.001, *** *p <* 0.0001.

It is important to note that HaCaT cells were affected only at higher doses of the compounds compared to SCC13. SCC13 cells were found to be most sensitive to **C278** treatment with an IC_50_ value of 0.50 ± 0.11 μM, which is almost half of the IC_50_ value of 1.15 ± 0.20 μM for HaCaT cells. A bar graph for HaCaT cell viability upon treatment with different doses of **C278** is presented in Figure 3B. Results of western blot experiments showed that the treatment of SCC13 cells with **C278** led to the downregulation in expression of the proliferating cell nuclear antigen (PCNA) in a dose-dependent manner as well (Figure 3C,D). These results suggest that **C278** is very effective in killing human skin cancer cells at a dose that is less toxic to a normal immortalized human keratinocytes.

### 2.2. Compound ***C278*** Treatment Restrains Cancer Cell Migration and Invasion in a Dose-Dependent Manner

**Figure 4:**
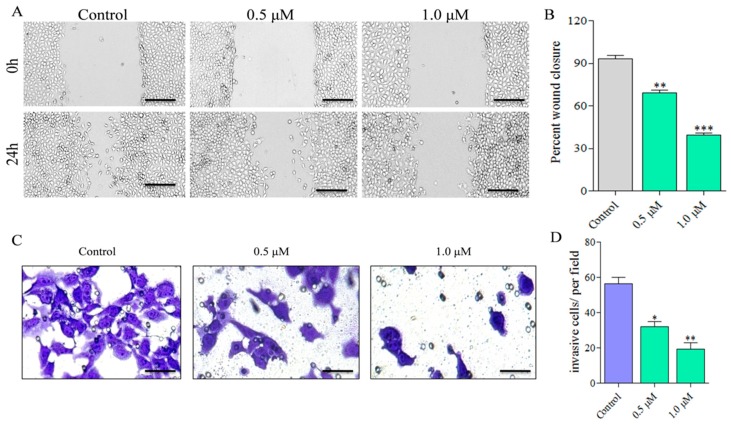
Effect of **C278** on migration and invasion ability of the SCC13 cells. (**A**) SCC13 cells were allowed to proliferate in a 24-well plate until reaching confluency. A scratch was made on the plate using a pipette tip. The unattached cells were washed off. The cells were then treated with either 0.1% DMSO or two doses of **C278** (0.5 µM and 1.0 µM) and incubated for 24 h. The images were captured at the time of drug treatment and after 24 h of incubation through a light microscope. (Scale bar: 200 µM). (**B**) Percent wound closure was measured using Image J software and plotted in bar graphs. Results are combined from two independent experiments and presented as mean ± SD; *n* = 4. (**C**) SCC13 cells were seeded in the invasion chambers and incubated for 4 h. Now the cells were treated either with 0.1% DMSO or two doses of **C278** (0.5 µM and 1.0 µM) for 24 h. The cells from the upper surface of the membrane were removed with cotton swabs. The invaded cells, which were in the lower surface of the membrane, were stained with crystal violet. Images of the invaded cells were captured using a light microscope. (**D**) The average number of cells invaded was counted in each group and plotted in bar graphs. Results are presented as mean ± SD; *n* = 4. * *p* < 0.01, ** p < 0.001, *** p < 0.0001.

Cancer cells have the ability to migrate and invade at distant sites in the body resulting in metastasis [33,34,35]. To examine whether **C278** treatment can inhibit the migration and invasion properties of SSC13 cells, we performed cell migration (wound healing) [36] and cell invasion (trans-well) assays [37,38]. A dose-dependent inhibition of the wound closure was observed as reflected in the micrographs (Figure 4A). Wound closure measurements showed that it decreased significantly to 69.06 ± 2.91% at 0.5 μM treatment which further decreased to 39.53 ± 2.17% at 1.0 μM treatment compared to 93.37 ± 3.35 percentage for the control group (Figure 4B). Similarly, through trans-well assay, we found that there was a dose-dependent decrease in the number of invasive cells as reflected in the micrographs (Figure 4C). Counting the number of the invasive cells showed that it decreased to 32.00 ± 4.24% at 0.5 μM treatment which further decreased to 19.50 ± 4.95% at 1.0 μM, compared to 56.50 ± 4.95% for the control group (Figure 4D). Together, these results indicate that **C278** inhibits the migratory and invasive ability of SCC13 cells in a dose-dependent manner.

### 2.3. Compound ***C278*** Induced Killing of SCC13 Cells Is Associated with the Modulation of Apoptosis and Autophagy Related Proteins

**Figure 5:**
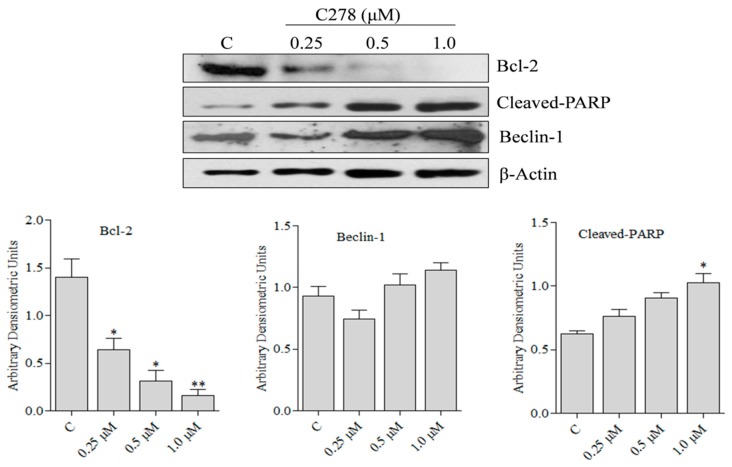
Effect of **C278** on apoptotic and autophagy related proteins in SCC13 cells. SCC13 cells were treated with 0.1% DMSO and 0.25, 0.5 and 1 µM of **C278** for 24 h. The cells were then lysed in cell lysis buffer and protein was estimated. Western blot was performed to estimate the level of expression for human Bcl2, Cleaved-PARP and Beclin-1. The expression of these proteins was normalized using β-Actin as a loading control. Figures are representatives of 2 independent experiments. Bands were analyzed densiometrically and bar graphs expressing arbitrary units are presented adjacent to the western blot. Error bar represents mean ± SD, *n* = 4. * *p* < 0.01. ** *p <* 0.001.

To determine if **C278** mediated killing of SCC13 cells involve the modulation in the expression of the pro- and anti-apoptotic genes as well as the activation of autophagy genes, we quantified the expression of the survival protein Bcl-2 [39], an apoptotic marker cleaved-PARP and an autophagy inducing protein beclin-1 through western blot. We found that **C278** significantly downregulated the expression of Bcl-2 protein in a dose-dependent fashion. Interestingly, the production of cleaved-PARP and the expression of beclin-1 increased in a dose-dependent manner (Figure 5). Taken together, these results suggest that **C278** mediated cell death in SCC13 cells, in part involves apoptosis and autophagy.

## 3. Discussion

Quinone-based compounds are known to exhibit potent activities against various human cancers [40,41]. In this study, we evaluated five quinone compounds that are analogs of marine natural products and elucidated their effects on a human non-melanoma skin cancer cell line SCC13 as well as an immortalized human skin cell line, HaCaT. We have also studied the mechanism of cell death in SCC13 cells upon the treatment with **C278**, which is most effective among the five compounds tested.

We found that SCC13 cells were more sensitive to **C278** treatment than the human keratinocyte, HaCaT. The dose of **C278** that was required to kill 50% of HaCaT cells was almost double the dose required to kill SCC13 cells. Based on the effect on the expression of the proliferation marker PCNA, we found that the proliferation of the cancer cells was also significantly inhibited upon **C278** treatment as indicated by the decreased expression of the PCNA. Previous studies have revealed that the PCNA positive cells decreased in number in tumor cells when treated with the quinone-based compounds [42].

Cancer cells are known to be highly proficient in migrating and invading to distant sites in the body [43]. We evaluated the effect of **C278** treatment on the migration and invasive ability of the SCC13 cells using a wound healing assay and a trans-well migration assay to find that **C278** not only significantly inhibited the migration of the SCC13 cells, it also inhibited the invasive ability of the cells as the number of cells that passed through the pores reduced significantly in the treated groups. Taken together, these results suggest that **C278** could not only inhibit cancer cell proliferation but may also inhibit its migratory and invasive abilities. However, it is unclear at this point if the migration and invasion inhibitory activities shown by **C278** are completely unrelated to cell killing and further studies are needed to establish this.

The Bcl-2 family of proteins plays an important role in the survival and in the apoptosis of the mammalian cells. The anti-apoptotic protein Bcl-2 inhibits the apoptosis, whereas the pro-apoptotic proteins promote apoptosis [44]. Our finding that **C278** significantly downregulated the anti-apoptotic protein Bcl-2 at the protein level indicates that **C278** mediated apoptosis in SCC13 cells involved reduction in the expression level of this protein. Additionally, we found that **C278** treatment induced production of cleaved-PARP, a well-known marker for apoptotic cells. Furthermore, it was observed that C278 treatment led to an increase in the expression level of autophagy inducing protein, beclin-1 [45]. Taken together, these results suggest that killing of SCC13 cells by **C278** is likely to be orchestrated by both apoptosis and autophagy related proteins.

## 4. Materials and Methods

### 4.1. Test Compounds

Five test compounds from the pyrroloiminoquinone family of marine natural products were evaluated for their ability to selectively kill non-melanoma skin cancer cell line SCC13 through MTT assay. The five compounds evaluated are **C278**, **C238**, **C11**, **C15** and **C45**. Their structures and chemical names are presented in Figure 2. We have previously evaluated these compounds (except **C278**) in breast [25,26] as well as lung cancer cells [46] and their general synthesis is reported in those publications. The IC_50_ and IC_90_ values of these five compounds against SSC13 as well as normal human keratinocyte HaCaT cells were determined as presented in Table 1. The most active and selective compound identified from this study is **C278**.

### 4.2. Synthesis of the Lead Compound ***C278***

**Figure 6:**
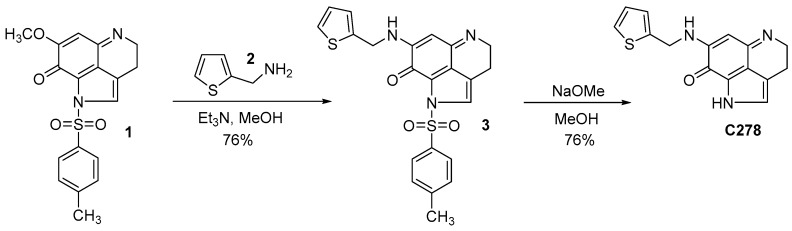
Synthesis of compound **C278**.

Synthesis of compound **C278** was carried out following a procedure previously reported [25,26] from our lab for the synthesis of other similar analogs in two steps starting from a key intermediate compound **1** as outlined in Figure 6. Compound **1** was synthesized in our lab following a previously reported procedure [47].

### 4.3. Amination of the Pyrroloiminoquinone Intermediate 1

To a solution of compound **1** (0.1 g, 0.21 mmol) in anhydrous MeOH (20 mL), a solution of thienyl amine, **2** (0.036 g, 0.26 mmol) in anhydrous MeOH (5 mL) was added dropwise taking 10 min. The reaction mixture was stirred at room temperature for 24 h. TLC examination (10% MeOH in CHCl_3_) indicated the completion of the reaction. The solvent was completely removed in vacuo and the residue obtained was dissolved in CHCl_3_ (10 mL) containing trifluoroacetic acid (0.1 mL). The solvent was completely removed again and the trifluoroacetate salt thus obtained was purified by column chromatography over Si gel using 3% MeOH in CHCl_3_ as eluent to afford the pure product **3** (0.058 g). This product was used in the next step without further purification and characterization.

### 4.4. Removal of the P-Toluene Sulfonyl Group from Compound 3 to Obtain ***C278***

To a solution of *N*-tosyl compound **3** (0.058 g) in anhydrous MeOH (20 mL), NaOMe (20 equiv.) was added and stirred at room temperature for 45 min. TLC analysis (5% MeOH in CHCl_3_) revealed the completion of the reaction. The resulting solution was cooled to 0 °C and quenched with TFA (30 equiv.) and stirred further at room temperature for 30 min. The solvent was removed in vacuo and the residue obtained was co-evaporated with CHCl_3_ (3 × 20 mL) to remove excess TFA. The crude product thus obtained was purified by column chromatography over Si gel using MeOH/CHCl_3_ (1:20) as eluent to obtain the pure detosylated compound **C278** (0.04 g, 48%); ^1^H NMR (CD_3_OD) δ 2.95 (t, 2H, *J* = 7.6 Hz), 3.85 (t, 2H, *J* = 7.6 Hz), 4.76 (s, 2H), 5.56 (s, 1H), 6.99 (dd, 1H, *J*_1_ = 5.1 Hz, *J*_2_ = 1.2 Hz), 7.10 (d, 1H, *J =* 2.7 Hz), 7.14 (s, 1H), 7.37 (dd, 1H, *J*_1_ = 5.1 Hz, *J*_2_ = 1.2 Hz); ^13^C NMR (CD_3_OD) δ 19.4, 43.0, 44.4, 86.5, 120.2, 123.6, 125.7, 126.9, 127.1, 127.9, 128.0, 139.6, 154.6, 160.1 and 168.8; MS (ES^+^) *m*/*z* 284 (M^+^). ^1^H-NMR, ^13^C-NMR and Mass spectrum of **C278** are available in the supplementary materials (Figure S1).

### 4.5. Chemicals and Reagents

3-(4,5-dimethylthiazol-2-yl)-2,5-diphenyl tetrazolium-bromide (MTT) was purchased from Sigma-Aldrich (St. Louis, MO, USA). The test compounds were dissolved in dimethyl sulfoxide (DMSO) (Sigma-Aldrich) to prepare a 10 mM stock solution, which was diluted in the medium to the required concentration for the assays. Dulbecco’s Modified Eagle Medium (DMEM), phosphate-buffered saline (PBS) and trypsin-EDTA were purchased from Corning, NY, USA. Fetal Bovine Serum (FBS), penicillin and streptomycin were procured from Gibco, Gaithersburg, MD, USA. All primary antibodies and the secondary antibodies were purchased from Santa Cruz Biotechnology, Santa Cruz, CA, USA.

### 4.6. Cell Line and Culture Conditions

SCC13 cells were purchased from ATCC and HaCaT cells were a kind gift from Dr. Andrzej Slominski’s (Professor, UAB department of Dermatology) laboratory where it is routinely cultured. SCC13 cells were derived from the epidermal keratinocytes from the human facial lesion [48]. SCC13 and HaCaT cells were cultured in DMEM supplemented with 10% FBS under humidified atmosphere of 5% CO_2_ at 37 °C. Cells were sub-cultured when they reached 70–80% confluency.

### 4.7. MTT Assay for Cell Viability

All cell lines were seeded on a 96-well cell culture plate at 2 × 10^5^ cells per well. After 24 h, the cells were treated with increasing doses of the compounds (0, 0.0625 μM, 0.125 μM, 0.25 μM, 0.5 μM, 1.0 μM). After incubation for 24 h, the medium was removed and 100 μL of MTT solution (500 µg/mL) in PBS was added and kept for 4 h at 37 °C. The formazan crystals thus formed were dissolved in DMSO and the optical density (OD) was measured at 570 nm using a microplate reader (BioTek Synergy H1 Plate Reader, Winooski, VT, USA).

### 4.8. Wound Healing Assay

SCC13 cells were seeded on 24-well cell culture plate at a density of 2 × 10^5^ cells/well and cultured until cells were confluent. The cells were scratched with the pipette tip and were gently washed once with PBS. The cells were then treated with **C278** at 0.5 µM and 1.0 µM of doses and incubated for 24 h in medium. Images were captured at 0 h and 24 h of drug treatment. The cell morphology was observed under inverted phase-contrast microscope (Keyence, BZ-X710, Osaka, Japan). Three fields in each well were photographed. Images were processed using Adobe Photoshop 5.5 (Adobe Systems, Inc., Mountain View, CA, USA) software. Percent change in wound area was measured by Image J software.

### 4.9. Cell Invasion Assay

For cell invasion assay, first Matrigel matrix coating of the invasion chambers (Falcon, Corning, Corning, NY, USA) was carried out for 2 h at 37 °C. Then SCC13 cells (5 × 10^4^) were seeded in the invasion chambers, which were kept in a 24-well cell culture plate. To the wells of the 24-well plate, 0.75 mL of medium supplemented with 5% FBS was added, which acts as a chemoattractant. The cells were incubated for 4 h, treated with different doses of **C278** and then incubated for 24 h. The non-invaded cells were removed by gently rubbing the top of the invasion chamber with a cotton swab. The cells were fixed in 4% paraformaldehyde for 10 min at room temperature, permeabilized with 100% methanol for 5 min, and then stained with crystal violet (0.05% *w*/*v*) for 20 min. The images were taken using an inverted microscope (Keyence, BZ-X710) and were processed using Adobe Photoshop.

### 4.10. Western Blotting

SCC13 cells (1–2 × 10^6^) cultured in 6-well culture plate were treated with different concentrations of **C278** for 24 h. Cells were then lysed in cell lysis buffer containing protease inhibitor cocktail and the protein concentrations in the cleared supernatants were estimated using the bicinchoninic acid (BCA) protein assay. The cell lysates were resolved by 10% SDS-PAGE and transferred to nitrocellulose membrane (BioRad, Hercules, CA, USA). The membranes were blocked with 5% bovine serum albumin (BSA) in tris-buffered saline (TBS) for 1 h at room temperature and probed with primary antibody for 2 h at a dilution recommended by the manufacturer. Membranes were then washed three times with wash buffer (TBS containing 0.5% Tween), and then incubated with horseradish peroxidase (HRP)-conjugated secondary Ab and detected by enhanced chemiluminescence (ECL) detection system according to the manufacturer’s instructions.

### 4.11. Statistical Calculations

Data is presented as the mean ± standard error of the mean. One-way analysis of variance (ANOVA) and Tukey’s multiple comparisons post-test were used for the analysis of data using Prism-Graph Pad version 5.0 (Graph pad Software, v.5.0, San Diego, CA, USA). Student’s *t*-test was employed to assess the statistical significance of differences between a pair of data sets. *p* < 0.05 was considered statistically significant.

## 5. Conclusions

In conclusion, we have evaluated the anticancer activities of five pyrroloiminoquinone marine natural product analogs against skin cancer using SCC13 cell line. Our results show that all five test compounds were effective in killing the SCC13 cells with compound **C278** being the most effective. Compound **C278** also inhibited cancer cell migration and invasion in a wound healing assay and Matrigel invasion assay. It is unclear at this point, if the migration and invasion inhibitory activities are completely unrelated to cell killing and further studies are needed to establish this. The expression of pro-apoptotic and autophagy proteins with concomitant downregulation in the expression of survival proteins were observed in **C278** treated cells. In summary, the marine natural product analog, compound **C278** showed promising anti-cancer activity against human skin cancer cells and holds potential to be developed as an effective anticancer agent to combat skin cancer. Future studies to demonstrate the in vivo activity of **C278** will further establish its role as a potent anti-skin cancer agent.

## Figures and Tables

**Table 1:** The inhibitory activity of five test compounds against SSC13 and HaCaT cell lines.

Compd	SSC13 Cells		HaCaT Cells	
	IC_50_ ± SD (μM)	IC_90_ ± SD (μM)	IC_50_ ± SD (μM)	IC_90_ ± SD (μM)
**C238**	0.82 ± 0.02	1.48 ± 0.04	2.35 ± 0.21	4.23 ± 0.36
**C278**	0.50 ± 0.11	0.90 ± 0.21	1.15 ± 0.21	2.07 ± 0.37
**C11**	0.51 ± 0.03	0.92 ± 0.06	0.48 ± 0.01	0.87 ± 0.03
**C15**	5.31 ± 0.19	9.56 ± 0.35	0.73 ± 0.09	1.32 ± 0.16
**C45**	2.58 ± 0.23	4.65 ± 0.42	5.24 ± 0.08	9.43 ± 0.15

Results are presented as the mean ± SD; *n* = 4.

## Supplementary Materials

The following are available online at https://www.mdpi.com/1660-3397/17/8/443/s1, Figure S1: ^1^H-NMR, ^13^C-NMR and Mass spectrum of **C278**.

## References

[1] Linares M.A., Zakaria A., Nizran P. (2015). Skin Cancer. Prim. Care.

[2] Lomas A., Leonardi-Bee J., Bath-Hextall F. (2012). A systematic review of worldwide incidence of nonmelanoma skin cancer. Br. J. Dermatol..

[3] Armstrong B.K., Kricker A. (2001). The epidemiology of UV induced skin cancer. J. Photochem. Photobiol. B.

[4] D’Orazio J., Jarrett S., Amaro-Ortiz A., Scott T. (2013). UV radiation and the skin. Int. J. Mol. Sci..

[5] Fabbrocini G., Triassi M., Mauriello M.C., Torre G., Annunziata M.C., De Vita V., Pastore F., D’Arco V., Monfrecola G. (2010). Epidemiology of skin cancer: Role of some environmental factors. Cancers.

[6] Simoes M.C.F., Sousa J.J.S., Pais A. (2015). Skin cancer and new treatment perspectives: A review. Cancer Lett..

[7] Yeong L.T., Abdul Hamid R., Saiful Yazan L., Khaza’ai H., Mohtarrudin N. (2015). Low dose triterpene-quinone fraction from Ardisia crispa root precludes chemical-induced mouse skin tumor promotion. BMC Complement. Altern. Med..

[8] Chinembiri T.N., du Plessis L.H., Gerber M., Hamman J.H., du Plessis J. (2014). Review of natural compounds for potential skin cancer treatment. Molecules.

[9] Yeong L.T., Hamid R.A., Yazan L.S., Khaza’ai H. (2013). Isolation of a quinone-rich fraction from Ardisia crispa roots and its attenuating effects on murine skin tumorigenesis. Asian Pac. J. Cancer Prev..

[10] Davids L.M., Kleemann B., Kacerovska D., Pizinger K., Kidson S.H. (2008). Hypericin phototoxicity induces different modes of cell death in melanoma and human skin cells. J. Photochem. Photobiol. B.

[11] Hill B.A., Kleiner H.E., Ryan E.A., Dulik D.M., Monks T.J., Lau S.S. (1993). Identification of multi-S-substituted conjugates of hydroquinone by HPLC-coulometric electrode array analysis and mass spectroscopy. Chem. Res. Toxicol..

[12] Wang J., Yi J. (2008). Cancer cell killing via ROS: To increase or decrease, that is the question. Cancer Biol. Ther..

[13] Blunt J.W., Copp B.R., Munro M.H., Northcote P.T., Prinsep M.R. (2005). Marine natural products. Nat. Prod. Rep..

[14] Blunt J.W., Copp B.R., Munro M.H., Northcote P.T., Prinsep M.R. (2006). Marine natural products. Nat. Prod. Rep..

[15] Haefner B. (2003). Drugs from the deep: Marine natural products as drug candidates. Drug Discov. Today.

[16] Proksch P., Edrada R.A., Ebel R. (2002). Drugs from the seas—Current status and microbiological implications. Appl. Microbiol. Biotechnol..

[17] Antunes E.M., Copp B.R., Davies-Coleman M.T., Samaai T. (2005). Pyrroloiminoquinone and related metabolites from marine sponges. Nat. Prod. Rep..

[18] Faulkner D.J. (2002). Marine natural products. Nat. Prod. Rep..

[19] Radisky D.C., Radisky E.S., Barrows L.R., Copp B.R., Kramer R.A., Ireland C.M. (1993). Novel cytotoxic topoisomerase II inhibiting pyrroloiminoquinones from Fijian sponges of the genus Zyzzya. J. Am. Chem. Soc..

[20] Carney J.R., Scheuer P.J., Kelly-Borges M. (1993). Makaluvamine G, a cytotoxic pigment from an an Indonesian Sponge *Histodermella* sp.. Tetrahedron.

[21] Fu X., Ng P.L., Schmitz F.J., Hossain M.B., van der Helm D., Kelly-Borges M. (1996). Makaluvic Acids A and B: Novel Alkaloids from the Marine Sponge Zyzzya fuliginosus. J. Nat. Prod..

[22] Utkina N.K., Makarchenko A.E., Denisenko V.A. (2005). Zyzzyanones B-D, dipyrroloquinones from the marine sponge Zyzzya fuliginosa. J. Nat. Prod..

[23] Utkina N.K., Makarchenko A.E., Denisenko V.A., Dmitrenok P.S. (2004). Zyzzyanone A, a novel pyrrolo[3,2-f]indole alkaloid from the Australian marine sponge Zyzzya fuliginosa. Tetrahedron Lett..

[24] Hu J.F., Schetz J.A., Kelly M., Peng J.N., Ang K.K., Flotow H., Leong C.Y., Ng S.B., Buss A.D., Wilkins S.P. (2002). New antiinfective and human 5-HT2 receptor binding natural and semisynthetic compounds from the Jamaican sponge Smenospongia aurea. J. Nat. Prod..

[25] Shinkre B.A., Raisch K.P., Fan L., Velu S.E. (2007). Analogs of the marine alkaloid makaluvamines: Synthesis, topoisomerase II inhibition, and anticancer activity. Bioorg. Med. Chem. Lett..

[26] Shinkre B.A., Raisch K.P., Fan L., Velu S.E. (2008). Synthesis and antiproliferative activity of benzyl and phenethyl analogs of makaluvamines. Bioorg. Med. Chem..

[27] Wang F., Ezell S.J., Zhang Y., Wang W., Rayburn E.R., Nadkarni D.H., Murugesan S., Velu S.E., Zhang R. (2010). FBA-TPQ, a novel marine-derived compound as experimental therapy for prostate cancer. Investig. New Drugs.

[28] Wang J., Lu Z., Wu C., Li Y., Kong Y., Zhou R., Shi K., Guo J., Li N., Liu J. (2019). Evaluation of the anticancer and anti-metastasis effects of novel synthetic sodium channel blockers in prostate cancer cells in vitro and in vivo. Prostate.

[29] Wang W., Qin J.J., Voruganti S., Nijampatnam B., Velu S.E., Ruan K.H., Hu M., Zhou J., Zhang R. (2018). Discovery and Characterization of Dual Inhibitors of MDM2 and NFAT1 for Pancreatic Cancer Therapy. Cancer Res..

[30] Wang W., Rayburn E.R., Velu S.E., Chen D., Nadkarni D.H., Murugesan S., Chen D., Zhang R. (2010). A novel synthetic iminoquinone, BA-TPQ, as an anti-breast cancer agent: In vitro and in vivo activity and mechanisms of action. Breast Cancer Res. Treat..

[31] Wang W., Rayburn E.R., Velu S.E., Nadkarni D.H., Murugesan S., Zhang R. (2009). In vitro and in vivo anticancer activity of novel synthetic makaluvamine analogues. Clin. Cancer Res..

[32] Xue B., Wang W., Qin J.J., Nijampatnam B., Murugesan S., Kozlovskaya V., Zhang R., Velu S.E., Kharlampieva E. (2017). Highly efficient delivery of potent anticancer iminoquinone derivative by multilayer hydrogel cubes. Acta Biomater..

[33] Kawahara E., Nakada N., Hikichi T., Kobayashi J., Nakanishi I. (2002). EGF and beta1 integrin convergently regulate migration of A431 carcinoma cell through MAP kinase activation. Exp. Cell Res..

[34] Krakhmal N.V., Zavyalova M.V., Denisov E.V., Vtorushin S.V., Perelmuter V.M. (2015). Cancer Invasion: Patterns and Mechanisms. Acta Naturae.

[35] Malliri A., Symons M., Hennigan R.F., Hurlstone A.F., Lamb R.F., Wheeler T., Ozanne B.W. (1998). The transcription factor AP-1 is required for EGF-induced activation of rho-like GTPases, cytoskeletal rearrangements, motility, and in vitro invasion of A431 cells. J. Cell Biol..

[36] Rodriguez L.G., Wu X., Guan J.L. (2005). Wound-healing assay. Methods Mol. Biol..

[37] Justus C.R., Leffler N., Ruiz-Echevarria M., Yang L.V. (2014). In vitro cell migration and invasion assays. J. Vis. Exp..

[38] Marshall J. (2011). Transwell(^®^) invasion assays. Methods Mol. Biol..

[39] Hata A.N., Engelman J.A., Faber A.C. (2015). The BCL2 Family: Key Mediators of the Apoptotic Response to Targeted Anticancer Therapeutics. Cancer Discov..

[40] Begleiter A. (1985). Studies on the mechanism of action of quinone antitumor agents. Biochem. Pharmacol..

[41] Lu J.J., Bao J.L., Wu G.S., Xu W.S., Huang M.Q., Chen X.P., Wang Y.T. (2013). Quinones derived from plant secondary metabolites as anti-cancer agents. Anti-Cancer Agents Med. Chem..

[42] Wen L., Lu X., Wang R., Jin X., Hu L., You C. (2018). Pyrroloquinoline quinone induces chondrosarcoma cell apoptosis by increasing intracellular reactive oxygen species. Mol. Med. Rep..

[43] Bozzuto G., Ruggieri P., Molinari A. (2010). Molecular aspects of tumor cell migration and invasion. Annali Dell'Istituto Superiore Sanitã.

[44] Tsujimoto Y. (1998). Role of Bcl-2 family proteins in apoptosis: Apoptosomes or mitochondria?. Genes Cells.

[45] Liang X.H., Jackson S., Seaman M., Brown K., Kempkes B., Hibshoosh H., Levine B. (1999). Induction of autophagy and inhibition of tumorigenesis by beclin 1. Nature.

[46] Nadkarni D.H., Wang F., Wang W., Rayburn E.R., Ezell S.J., Murugesan S., Velu S.E., Zhang R. (2009). Synthesis and in vitro anti-lung cancer activity of novel 1, 3, 4, 8-tetrahydropyrrolo [4, 3, 2-de]quinolin-8(1H)-one alkaloid analogs. Med. Chem..

[47] Sadanandan E.V., Pillai S.K., Lakshmikantham M.V., Billimoria A.D., Culpepper J.S., Cava M.P. (1995). Efficient Syntheses of the Marine Alkaloids Makaluvamine D and Discorhabdin C: The 4,6,7-Trimethoxyindole Approach. J. Org. Chem..

[48] Rubin A.L., Rice R.H. (1986). Differential regulation by retinoic acid and calcium of transglutaminases in cultured neoplastic and normal human keratinocytes. Cancer Res..

