# Supplementary material for: A Novel Marine Natural Product Derived Pyrroloiminoquinone with Potent Activity against Skin Cancer Cells
Source: Mar Drugs. 2019 Jul 27;17(8):443. doi: 10.3390/md17080443 (PMC6722685; doi:10.3390/md17080443)

Supporting Information

A novel marine natural product derived pyrroloiminoquinone with potent activity against skin cancer cells

Jaden Cowan [1,#], Mohammad Shadab [#,2], Dwayaja H. Nadkarni, Kailash K C, Sadanandan E. Velu [*,1] and Nabiha Yusuf [*,2,3]

[1] Department of Chemistry, University of Alabama at Birmingham, 901, 14th Street S, Birmingham, AL-35294, USA
[2] Department of Dermatology, University of Alabama at Birmingham, 1670 University Boulevard, VH566A
[3] Veteran Affairs Medical Center, Birmingham, AL-35294, USA

[#] Both authors contributed equally to this manuscript
* Joint Corresponding Authors: Nabiha Yusuf, PhD, MSPH, Associate Professor, Department of Dermatology, University of Alabama at Birmingham, 1670 University Boulevard, VH 566A, PO Box 202, Birmingham, Alabama 35294-0019; Tel.: (205) 934-7432, Fax: (205) 934-5745; E-mail: nabihayusuf@uabmc.edu; Sadanandan E. Velu, PhD, Associate Professor, Department of Chemistry, CHEM 280, University of Alabama at Birmingham, 901, 14th Street S, Birmingham, AL 35294-1240, USA, Tel: (205) 975 2478 ; Fax: (205) 934-2543; Email: svelu@uab.edu

Content: ^{1}H-NMR, ^{13}C-NMR and Mass spectrum of **C278**.

^{1}H NMR of Compound **C278**

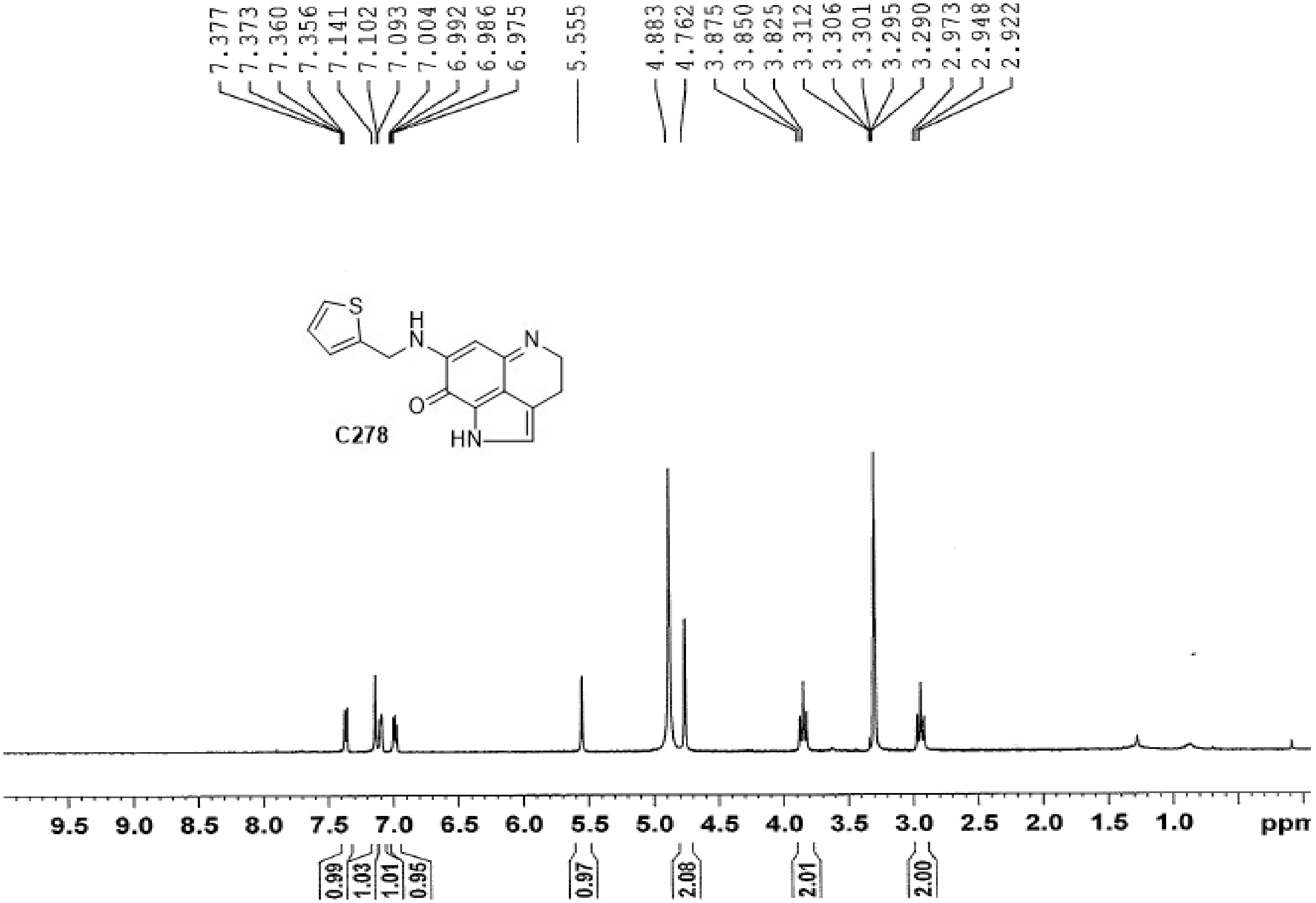

^{13}C-NMR of Compound **C278**

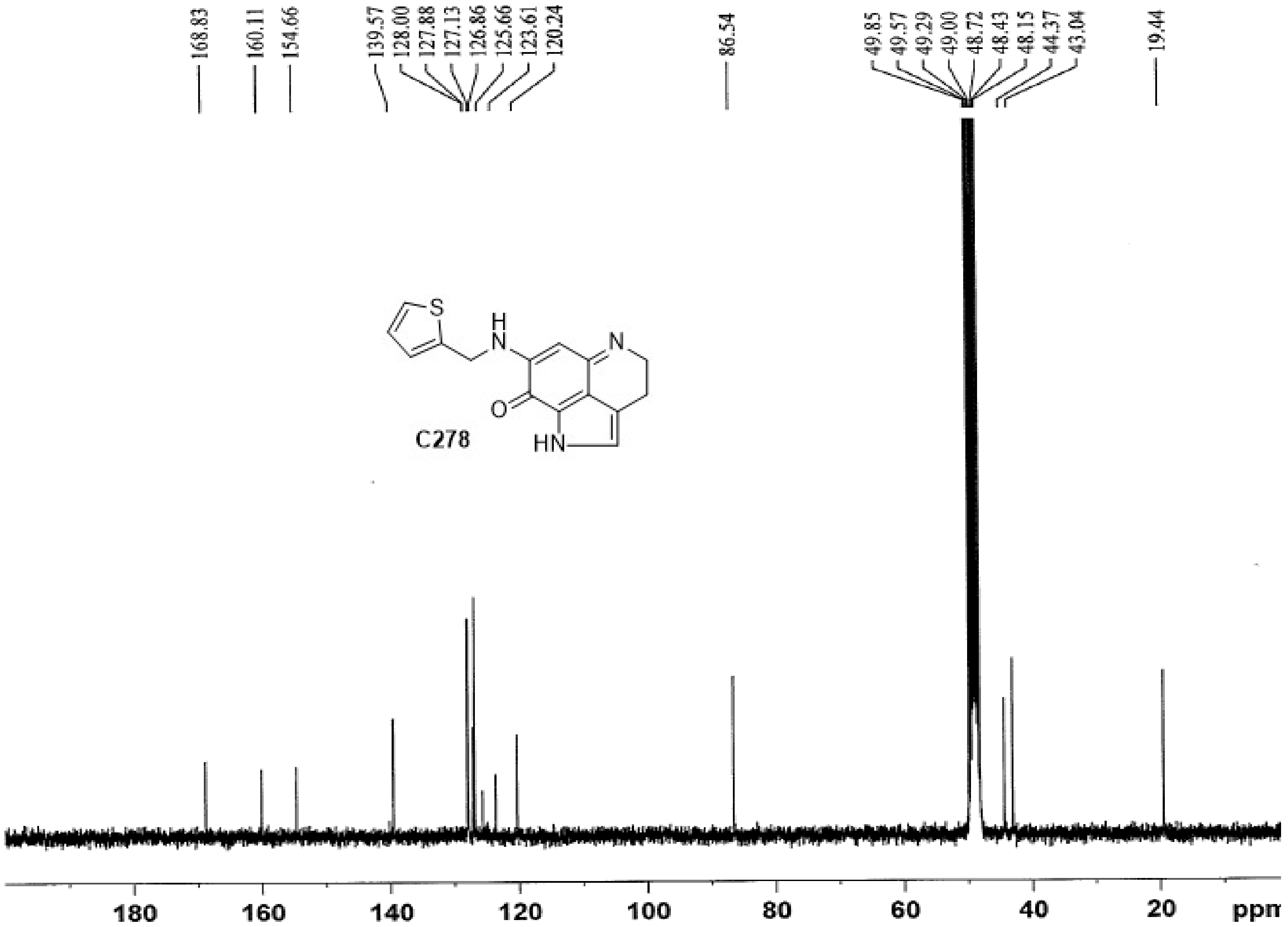

File ..7-19\160719-L_175-1000DWB 220-420NM-28399.D Tgt Mass (EZX):

Injection Date	: 16-Jul-19, 16:29:57	Seq. Line	: 0
Sample Name	: JC-83	Location	: P1-F-04
Acq. Operator	: Syed Kaleem Ahmed	Inj	: 0
Spec. Reported	: MS Integration	Inj Volume	: 1 ul
Acq. Method	: C:\Chem32\2\METHODS\L_175-1000DWB 220-420NM.M		
Analysis Method	: C:\Chem32\2\METHODS\L_175-1000DWB 220-420NM.M		

Sample Info : Easy-Access Method: 'HMW General Loop 175-1000D'

Method Info : Solvent 50:50 H2O:MeOH
0.1% FA
no column

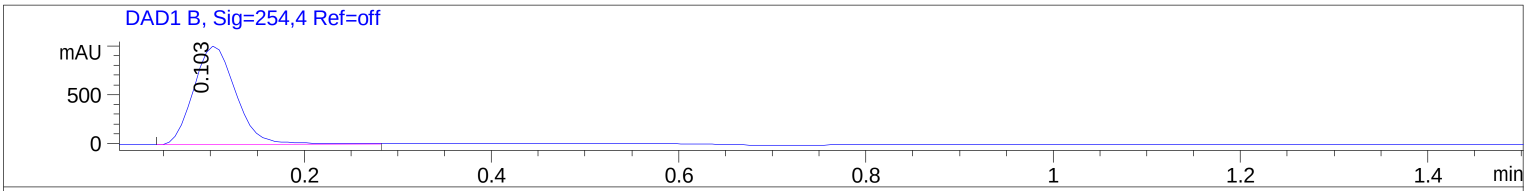

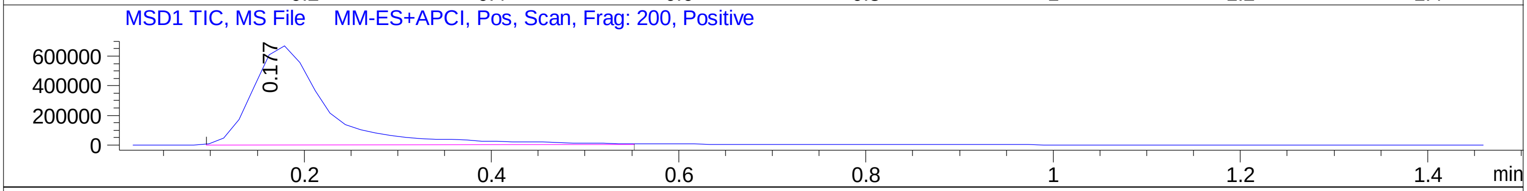

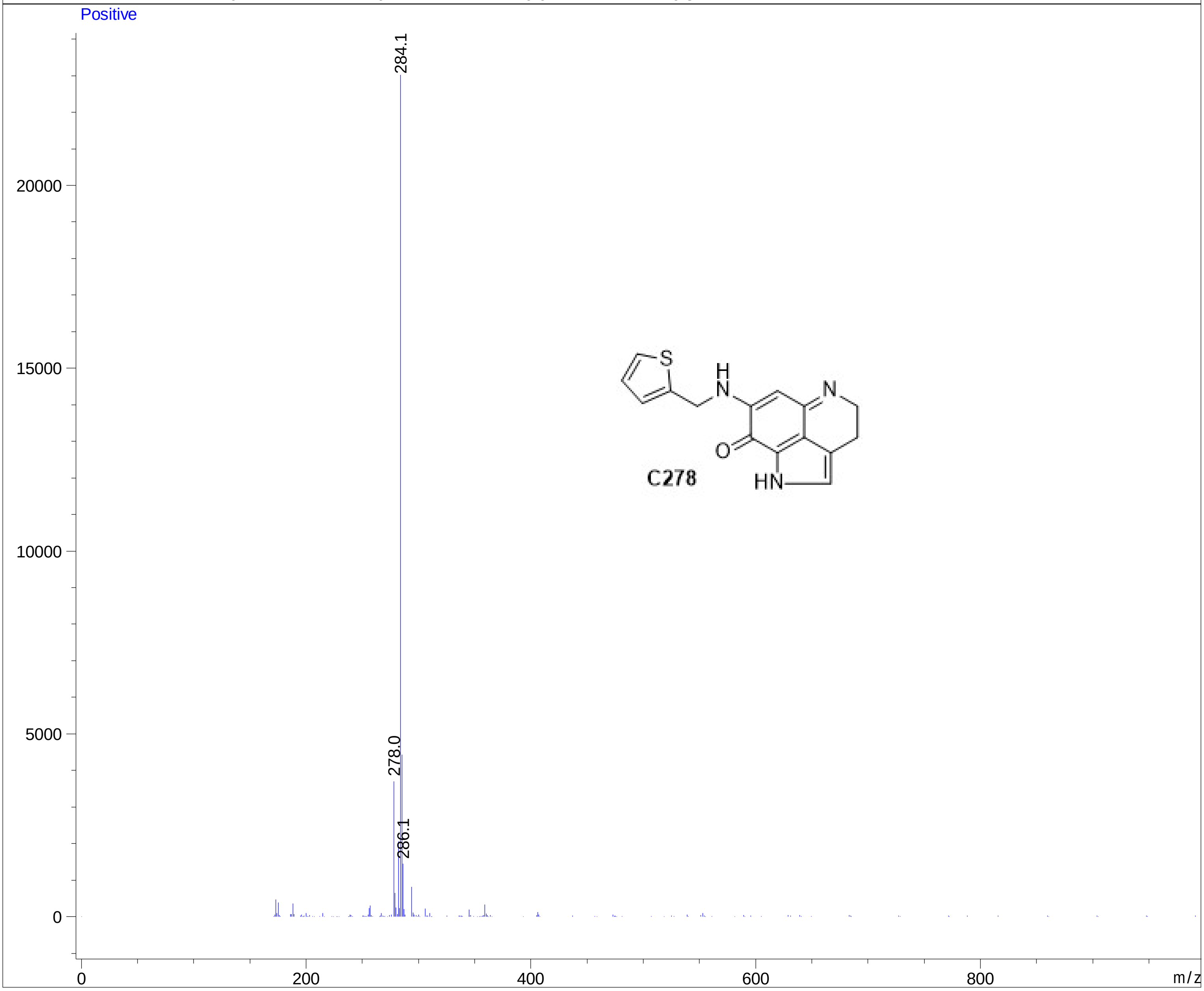

Supplement: Supplementary file 1 [file marinedrugs-17-00443-s001.pdf]
